# Risk Stratification in Renal Cell Carcinoma: A Narrative Review

**DOI:** 10.3390/cancers18132081

**Published:** 2026-06-26

**Authors:** Nykiera Dixon, Vivian Wong, Fuat Bicer, Shawn Dason, Eric A. Singer

**Affiliations:** 1 Division of Urologic Oncology, The Ohio State University Comprehensive Cancer Center, Columbus, OH 43210, USA; nykiera.dixon@osumc.edu (N.D.);; 2 Division of Medical Oncology, The Ohio State University Comprehensive Cancer Center, Columbus, OH 43210, USA

**Keywords:** renal cell carcinoma, metastatic renal cell carcinoma, risk stratification, localized renal cell carcinoma, risk categorization

## Abstract

Renal cell carcinoma (RCC) accounts for the majority of kidney cancers, with approximately 80,000 new diagnoses and over 14,000 deaths annually in the United States. Risk stratification is essential for prognostication, treatment selection, and clinical trial design across all disease stages. In localized and locally advanced RCC, pathological stage, histology, and grade remain the primary prognostic factors, while the International Metastatic Renal Cell Carcinoma Database Consortium (IMDC) criteria serve as the standard risk stratification tool in the metastatic setting. However, current models rely predominantly on clinical and pathologic variables that act as indirect surrogates of tumor biology and do not account for the molecular heterogeneity inherent to RCC. This narrative review synthesizes and compares established and emerging risk stratification and prognostic models across all stages of RCC. Established models such as the IMDC criteria and the stage, size, grade, and necrosis (SSIGN) score demonstrate robust prognostic performance but are limited by their reliance on clinical and pathologic variables alone. Emerging biomarkers—including circulating tumor DNA, methylated DNA, artificial intelligence-based radiomics, and tissue-based molecular signatures—show promise for improving risk discrimination. The molecular heterogeneity of RCC underscores an urgent need for integrated molecular–clinical–pathologic prognostic tools tailored to specific histologic subtypes to enable more precise, individualized care.

## Simple Summary

Kidney cancer, specifically renal cell carcinoma, affects about 80,000 Americans each year and causes over 14,000 deaths. Doctors currently predict how the disease will behave using tools that look at tumor size, stage, and blood test results, but these methods do not account for the unique genetic makeup of each person’s cancer. This research reviewed both these existing tools and promising newer approaches—such as blood tests that detect tumor DNA fragments, artificial intelligence analysis of medical images, and molecular profiling of tumor tissue. The goal is to combine traditional clinical information with these advanced techniques to create more personalized prediction tools, helping doctors better determine which patients need aggressive treatment and which may benefit from less intensive care.

## 1. Introduction

Renal cell carcinoma (RCC) accounts for the majority of kidney cancers and is broken down into multiple subtypes, with clear cell being the most common [1]. In the United States alone, 80,000 people are diagnosed with RCC each year and the disease accounts for over 14,000 deaths annually [2].

The main prognostic factors in localized and locally advanced RCC are pathological stage, histology, and grade [3]. These factors are also used in a predictive manner, such as in selection for adjuvant systemic therapy following surgical resection. In the metastatic setting, the International Metastatic Renal Cell Carcinoma Database Consortium (IMDC) criteria comprise the main risk stratification tool used for both prognostic and predictive purposes.

Recent work in risk stratification has focused on improving models by incorporating factors beyond the traditional patient and pathologic factors, including molecular characteristics that reflect tumor biology. Despite the expansion of these methods and more reliable ways to compare them, there are many gaps in knowledge amongst these emerging tools and no comprehensive review of current RS models. To address this, the present review will synthesize and compare established and emerging risk stratification and prognostic models for localized, locally advanced, and metastatic RCC.

## 2. C Indexes

The performance of risk stratification models is evaluated using the concordance index (C-index) which quantifies a model’s discriminative ability by measuring the probability that, for any randomly selected pair of patients, the model correctly predicts which patient will experience the event first.

## 3. Anatomic/Morphologic Prognostic Models

### 3.1. Tumor, Node, Metastasis Staging System

The American Joint Committee on Cancer (AJCC)’s Tumor, Node and Metastasis (TNM) staging system is the original risk stratification system used in RCC [4]. Since its first publication in 1977, it has undergone several revisions with the most recent being in 2018. TNM staging is essential for prognosis and treatment, especially for surgical planning. Table 1 shows comparisons of the most recent AJCC breakdown of TNM staging as it relates to RCC [5,6,7].

### 3.2. International Society of Urologic Pathology (ISUP) Grading System for RCC

Another important tumor characteristic used to classify RCC is the International Society of Urological Pathology (ISUP) Grading System for RCC [8] which replaced the older Fuhrman system [9]. This system is a 4-tier graded system recommended for ccRCC and pRCC with Grades 1–3 indicating increasingly conspicuous nucleoli and Grade 4 defined with high-grade features including sarcomatoid or rhabdoid differentiation, tumor giant cells or extreme nuclear pleomorphism [8].

### 3.3. American Urologic Association (AUA) Risk Stratification for Renal Cancers

In 2021, the AUA released guidelines stratifying kidney cancers to low, intermediate, high, or very high risk based on pathologic review using TNM staging and tumor grade following partial or radical nephrectomy [10] (Table 2). Patients with pT1 and Grade 1/2 are classified low risk10. pT2 and Grade 3/4, or pT2 with any grade is intermediate risk10. pT3 with any grade is high risk, and pT4 or pN1, sarcomatoid or rhabdoid differentiation, or macroscopic positive margin is very high risk [10] (Table 2). Recurrence rates for low-risk disease range from 6.4 to 15.4%, for intermediate-risk disease range from 20 to 32% and for high-risk disease near 50% [11] (Table 2). For very high-risk disease, recurrence rates are over 65% [12]. Specifically, sarcomatoid differentiation could have over a 70% recurrence rate [13] (Table 2).

In early 2026, Garofano et al. performed a retrospective multicenter study of 5711 patients with surgically treated nonmetastatic clear cell renal cell carcinoma (ccRCC) to compare the prognostic performance of the American Urological Association (AUA) risk stratification system with the KEYNOTE-564 trial eligibility criteria [15]. The results demonstrated that the AUA model showed superior discrimination for recurrence-free survival (RFS) compared to KEYNOTE-564 (C-index 0.71 vs. 0.68; *p* = 0.03), with progressively higher recurrence risk in intermediate- (HR 2.38), high- (HR 2.92), and very high-risk groups (HR 3.68) compared to low-risk patients (all *p* < 0.001) [15]. For OS, both models showed identical discrimination (C-index 0.73; *p* = 0.67), while for CSS, discrimination was similar (AUA 0.79 vs. KEYNOTE-564 0.78; *p* = 0.65) [15]. The authors concluded that the AUA classification’s superior performance for RFS and improved reclassification compared to KEYNOTE-564 likely stems from its inclusion of adverse pathologic features such as sarcomatoid/rhabdoid differentiation and positive surgical margins, which were not part of the KEYNOTE-564 eligibility criteria [15].

### 3.4. Mayo Clinic Validation of AUA Risk Groups

In 2024, Zganjar et al. validated AUA risk stratification groups for RCC. The study analyzed 3191 patients with clear cell RCC and 633 patients with papillary RCC from the Mayo Clinic Nephrectomy Registry (1980–2012) who underwent radical or partial nephrectomy for unilateral, M0 disease [16]. In validating the AUA risk model, the authors compared it to the Mayo institutional model. This model is a histology-specific prognostic tool that helps predict PFS and CSS after surgical resection of metastatic disease. For clear cell RCC, the AUA risk groups achieved C-indexes of 0.780 for progression-free survival and 0.811 for cancer-specific survival compared to 0.815 and 0.857, respectively, for the Mayo institutional model (both *p* < 0.001) [16]. For papillary RCC, the AUA system performed comparably to the Mayo model, with C-indexes of 0.775 versus 0.751 for PFS (*p* = 0.002) and 0.830 versus 0.803 for cancer-specific survival (CSS) (*p* = 0.2) [16]. Overall, authors concluded that AUA risk groups are practical and effective tools for categorizing RCC patients; however, there is more prognostic value for histologic-specific models, especially when considering clear cell histology.

While the AUA risk stratification system demonstrates robust discriminatory ability with C-indexes of approximately 0.76–0.83 for PFS, RFS and CSS across different RCC subtypes [15,16,17], the traditional TNM staging system upon which it is partially based has inherent limitations that underscore the ongoing need for more comprehensive prognostic tools. These limitations include: limited prognostic factors, lack of histology-specific prognostication, heterogeneity within stage groups, grouping together tumors with perirenal fat invasion, renal vein invasion, renal sinus invasion, and collecting system invasion despite potentially different prognoses [18], amongst others. In 2019, Patel et al. highlighted the substantial expansion of kidney cancer therapies, contrasted with the limited progress in RCC prognostic systems, largely underscoring the need to better align stage groupings with patient outcomes in order to improve prognostic accuracy [19].

Similarly, Srivastava et al. showed that patients diagnosed with lymph node-positive RCC, pT1-3N1M0, have survival outcomes similar to those with metastatic (stage IV) RCC [20]. Using data from the National Cancer Database, the study compared three groups: lymph node-negative stage III (pT3N0M0), lymph node-positive stage III (pT1-3N1M0), and stage IV metastatic (pT1-3N0M1) RCC [20]. The results showed that 5-year survival rates for lymph node-positive stage III RCC (22.7%, 95% CI, 20.6–24.9%) were nearly identical to those for stage IV metastatic disease (15.6%, 95% CI, 11.1–23.8%) and much worse than for lymph node-negative stage III RCC (61.9%, 95% CI, 60.3–63.4%) [20]. The authors ultimately recommended re-classifying lymph node-positive, non-metastatic RCC as stage IV, improving prognostication, patient counseling, and selection for adjuvant therapy and clinical trials (Table 3).

### 3.5. R.E.N.A.L Nephrometry Score

The RENAL nephrometry score is another tool utilized to stratify anatomic complexity of renal masses, primarily to assist with surgical planning and help predict perioperative and oncologic outcomes [23]. It was first introduced in 2009 by Drs. Kutikov and Uzzo [23]. It examines five objective factors: radius, exophytic vs. endophytic, nearness to the collecting system, anterior vs. posterior location, and location relative to polar lines. Each score is summed and tumors are categorized into low, intermediate and high risk, with high scores being associated with more aggressive pathology, increased risk of complications and greater likelihood of requiring radical rather than partial nephrectomy [23]. Nephrometry scores provide standardized, reproducible assessment of renal masses and aid in surgical decision making. The score used in conjunction with other risk stratification tools, such as TNM staging, contribute to overall clinical decision making in regard to treatment and surgical management [23]. Studies have shown concordance rates for each individual component of the RENAL (hilar) nephrometry score to be 0.96, 0.92, 0.86, 0.96, 0.89, and 0.99, respectively [24].

However, there are several limitations with the RENAL nephrometry score. In a large external validation cohort of 1129 patients with cT1 renal masses, Koo et al. found the nomogram’s ability to predict malignancy was moderate (AUC 0.72 for cT1 tumors), but its performance for predicting high-grade RCC was poor (AUC 0.57 for cT1 tumors and 0.50 for cT1a tumors) compared to the original development cohort (AUC 0.73) [25]. The authors ultimately concluded that the RENAL nephrometry score nomogram had limited accuracy for predicting high-grade renal cell carcinoma (RCC) in solid, enhancing, and small renal masses, especially in small (cT1) tumors [25].

### 3.6. Stage, Size, Grade and Necrosis (SSIGN) Score

The Mayo Clinic’s tumor stage, size, grade, and necrosis (SSIGN) score provides a risk stratification tool that was originally validated with patients who underwent radical nephrectomy for ccRCC between 1970 and 1998 [26]. This stratification tool aimed at predicting cancer-specific survival and incorporates tumor (TNM) stage, size, nuclear grade, and presence of tumor necrosis in histopathologic examination. Additional pathologic features examined included sarcomatoid features, cystic architecture, multifocality and status of surgical margins [26]. Although clinical features including age, smoking history, and ECOG status were evaluated, these ultimately were not added to the prognostic model as these were not significantly associated with death from ccRCC [26].They found that TNM stage, tumor size greater than 5.0 cm, nuclear grade and histological tumor necrosis were all significantly associated with ccRCC-specific mortality for use in the prognostic model (*p* < 0.001) [26].

A point system was given to each category and then added to form a total [26]. For T Stage: pT1 = 0, pT2 = 1, pT3a-c = 2, pT4 = 0. For N staging: pNx/0 = 0 and pN1/2 = 2. For M stage: pM0 = 0 and pM1 = 4. For tumor size: less than 5cm = 0 and 5 cm or greater = 2. For nuclear grade: 1/2 = 0 and for 3/4 = 3. For necrosis: absent = 0 and present = 2. These scores are added and the percent estimated 5-year cancer-specific survival are listed: 0–1 = 99.4% (SE 0.4), 2 = 94.8% (SE 1.5), 3 = 87.8% (SE 2.5), 4 = 79.1% (SE 3.1), 5 = 65.4% (SE 4.1), 6 = 54.0 (SE 5.6), 7 = 41.0 (SE 3.8), 8 = 23.6% (SE 4.3), 9 = 19.6% (SE 4.3), and 10 or greater 7.4 (SE 2.4) [26]. The SSIGN score has been validated in many studies with C-indexes ranging from 0.81 to 0.90 [27,28,29,30].

Despite these robust C-index values demonstrating excellent discriminatory ability, the SSIGN score has important limitations that may affect its clinical utility, particularly its exclusion of patient-specific factors, such as performance status and comorbidities, and its binary classification of tumor necrosis as simply present or absent rather than quantifying the extent of necrosis. Additionally, the binary classification of tumor necrosis does not help to delineate whether degree of tumor necrosis correlates with better prognosis. The inverse question is also not answered. This further stratification could help provide additional prognostic information. Studies such as the retrospective evaluation of 318 ccRCC cases by Syed et al. provide evidence that percentage of necrosis is associated with metastasis or recurrence with group 2 (1–10% necrosis) having adjusted odds ratio (AOR) of 8.72 (95% CI 3.62–20.98) and group 3 (>10% necrosis) having AOR of 9.48 (95% CI 3.96–22.18) as compared to group 1 (0% necrosis) [31].

### 3.7. Karakiewicz Prognostic Nomogram

In 2007, Karakiewicz et al. demonstrated that a prognostic nomogram incorporating seven clinicopathologic variables achieves superior accuracy for predicting cancer-specific survival in renal cell carcinoma compared to conventional staging systems, with external validation showing predictive accuracies exceeding 86% across multiple time points [32]. The study utilized two large cohorts, a development cohort of 2530 patients and an external validation cohort of 1422 patients who underwent radical or partial nephrectomy for renal cortical tumors [32]. The nomogram integrated 2002 TNM stage, tumor size, Fuhrman grade, histologic subtype, presence of local symptoms, age, and sex as predictor variables identified through Cox proportional hazards regression [32]. External validation demonstrated exceptional predictive accuracy, with concordance indexes of 87.8%, 89.2%, 86.7%, and 88.8% at 1, 2, 5, and 10 years after nephrectomy, respectively [32]. When compared directly to an established staging scheme, the nomogram showed statistically significant improvements: at 2 years, the nomogram achieved 89.2% accuracy compared to 86.1% for the staging system (*p* = 0.006); and at 5 years, 86.7% versus 83.9% (*p* = 0.02) [32].

## 4. Integrated Clinicopathologic Prognostic Models

### 4.1. University of California Los Angeles Integrated Staging System (UISS)

In efforts to improve risk stratification models, some authors have incorporated clinical features in addition to the solely anatomic systems like TNM staging and RENAL score. One such system, the University of California Los Angeles Integrated Staging System (UISS) incorporates the 1997 tumor–node–metastasis (TNM) stage, Fuhrman grade, and Eastern Cooperative Oncology Group (ECOG) performance status (PS) and stratifies survival for patients with RCC [33]. Patients are first categorized into non-metastatic and metastatic. When the system was first described in 2001, there were five risk categories ranging from I toV. These risk categories were then converted to risk groups, low risk (LR), intermediate risk (IR) and high risk (HR) in two categories of RCC, non-metastatic and metastatic [34]. The 5-year disease-specific survival in patients with non-metastatic RCC low risk was 91.1% (SE 3.6), intermediate risk was 80.4 (SE 4.0) and high risk was 54.7% (SE 5.4) [34]. The 5-year disease-specific survival in patients with metastatic RCC low risk was 32.0% (SE 8.7), intermediate risk was 19.5 (SE 3.2) and high risk was 0% [34]. One international validation study of 4202 patients showed that for non-metastatic RCC, the 5-year overall survival rates were 92%, 67%, and 44% for LR, IR, and HR, respectively (*p* < 0.001). The 3-year OS was 37%, 23%, and 12% for LR, IR, and HR, respectively (*p* < 0.001) [35].

While the original 2002 article did not report C-index values, subsequent external validations have consistently shown C-index values ranging from 0.68 to 0.86, with most studies reporting values between 0.72 and 0.84, demonstrating good to excellent discriminatory ability for predicting survival outcomes in RCC patients [36,37,38,39]. The UISS has been validated across histologic subtypes as well as in localized, advanced and metastatic RCC and cytoreductive nephrectomy populations, still with comparable C-indexes to other validated risk stratification tools. These values confirm its utility as a reliable, clinically accessible tool for risk stratification, patient counseling, and clinical trial design.

### 4.2. Kattan et al. [40] Tool for Treatment Failure Predictions

Similar to the UISS risk tool, in 2001, Kattan et al. presented a validated statistical tool that predicted the 5-year probability of treatment failure (recurrence or death from disease) following nephrectomy for renal cell carcinoma, demonstrating good discriminatory ability with an area under the receiver operating characteristic curve of 0.74 [40]. They analyzed 601 patients who underwent nephrectomy and were the first to incorporate patient symptoms (incidental, local, or systemic) to their model, which also used histologic subtype (chromophobe, papillary, or conventional), tumor size, and pathological stage [40]. Disease recurrence occurred in 66 of 601 patients (11%), with a median follow-up of 40 months and maximum follow-up of 123 months for patients without treatment failure [40]. The 5-year probability of freedom from failure for the entire cohort was 86% (95% confidence interval of 82–89%) [40].

### 4.3. Paraneoplastic and Symptomatic (PRIMAL) Score

An emerging model, the paraneoplastic and symptomatic (PRIMAL) score is another prognostic tool used in patients with RCC that integrates clinical presentation, abnormal laboratory values, and paraneoplastic syndromes. In 2025, Musso et al. analyzed 5256 T1-T4, N0/1, M0/1 RCC surgical patients from four institutions in a retrospective study [41]. Preoperative values included hematuria, visceral pain, nausea/vomiting, platelets <100 × 109/L, hypoalbuminemia (<3.5 g/dL), anemia (<11.5 mg/dL for women, <12.5 mg/dL for men), elevated De Ritis Ratio (AST/ALT > 1.25), and elevated neutrophil-to-lymphocyte-ratio (NLR > 2.27)39. Patients were stratified into risk categories based on the number of aforementioned criteria they met. Risk categories included low (0 variables), favorable-intermediate (1–2), unfavorable-intermediate (1–2 + anemia), and high (≥3). They found that of the 5256 patients analyzed, 2513 (48%) patients had low, 1532 (29%) favorable-intermediate, 909 (17%) unfavorable-intermediate, and 302 (6%) high PRIMAL score with high PRIMAL score exhibiting high hazard ratios for all-cause mortality (ACM) (HR = 7.71, 95% CI = 5.98–9.93) and cancer-specific mortality (CSM) (HR = 8.54, 95% CI = 5.96–12.24) [41]. Five-year OS rates were 91%, 82%, 65% and 46%, while CSS rates were 95%, 90%, 76% and 60% for the low, favorable-intermediate, unfavorable-intermediate and high groups, respectively [41]. PRIMAL incorporates both the clinical symptoms at time of presentation as well as the laboratory values and serves as a comprehensive risk stratification tool that aids in initial assessment and guides management decisions.

### 4.4. Stage, Sarcomatoid, PBRM1 Expression and Necrosis (SSPN) Score

Similarly, the SSPN score is a novel prognostic scoring system that integrates clinicopathologic factors with PBRM1 immunohistochemical expression to predict postoperative recurrence in patients with non-metastatic clear cell renal cell carcinoma. The SSPN score stratifies patients into four distinct risk groups based on the number of adverse factors present, low risk (0 factors), intermediate risk (one factor), high risk (2–3 factors), and very high risk (four factors), with significant differences in RFS among groups (*p* < 0.001) [42]. The study retrospectively analyzed 389 non-metastatic ccRCC patients who underwent radical surgery between 2006 and 2017, with a median follow-up of 61 months during which 53 patients (13.6%) experienced recurrence [42]. Multivariable analysis identified four independent factors associated with shorter RFS: ≥pT3 stage (HR 3.64), sarcomatoid or rhabdoid component (HR 3.29), PBRM1 negativity (HR 3.39), and tumor necrosis (HR 3.60) (all *p* ≤ 0.005) [42]. The model demonstrated a C-index of 0.841 for 5-year RFS prediction [42].

### 4.5. ASSURE Nomogram

Likewise, the ASSURE nomogram is a contemporary prognostic model developed using prospective data from the ECOG-ACRIN 2805 (ASSURE) trial. The study analyzed 1735 patients with high-risk localized and locally advanced RCC over a median follow-up of 9.6 years, during which 887 DFS events occurred. The model incorporated five common tumor variables—histology, size, grade, tumor necrosis, and nodal involvement—with tumor histology emerging as the single most powerful predictor for each outcome. The model predicted three clinically relevant outcomes: DFS, OS, and early disease progression (EDP). Performance characteristics showed C-statistics of 78.4% and 81.9% at 1 year for DFS and OS, respectively, though discriminatory ability declined over time to a global C-index of 0.68 for DFS and 0.69 for OS. The EDP model achieved a C-index of 75.1%. Importantly, this model is histology-inclusive and incorporates TNM staging, providing added flexibility compared to traditional staging-based approaches. The ASSURE nomogram demonstrates superior predictive accuracy compared to existing retrospective models for patients with high-risk renal cell carcinoma after nephrectomy. The authors demonstrated that it can inform treatment protocols, clinical trial eligibility, and patient counseling for high-risk RCC patients after surgical resection [43].

## 5. Non-Clear Cell RCC: Anatomic and Clinicopathologic Prognostic Models

### 5.1. Venous Tumor Thrombus, Nuclear Grade, Size, T and N Stage (VENUSS) Model

One of the few histopathologically validated prognostic model specific for pRCC is the VENUSS model (VEnous tumor thrombus, NUclear grade, Size, T and N Stage) published in 2019 [44]. Data was collected from the multi-institutional prospective ASSURE clinical trial and 150 high-risk pRCC patients were evaluated for this model [44]. A score is assigned to the variables listed in the name of the model: venous tumor thrombus (absent = 0 pts, present = 2 pts), nuclear grade (G1 or G2 = 0 pts, G3 or G4 = 2 pts), tumor size (≤4 cm = 0 pts, ≥4.1 cm = 2 pts), T stage (pT1 = 0 pts, pT2 = 1 pts, pT3/pT4 = 2 pts), and N stage (pNx/pN0 = 0 pts, pN1 = 1 pts) [44]. Patients with 0–2 points are assigned into the low-risk group, 3–5 points into the intermediate-risk group, and ≤6 points into the high-risk group [44]. This study analyzed 556 patients and found the 5-year cumulative incidence of recurrence ranged from 2.9% (95% CI = 1.9–3.9%) in the low-risk group to 54.5% (95% CI = 47.3–61.8%) in the high-risk group [44]. Specifically, this study found the cumulative incidence of recurrence was 5.9% (95% CI = 4.9–6.9%) at 1 year, 9.0% (95% CI = 7.7–10.2%) at 2 years and 11.7% (95% CI = 10.2–13.2%) at 5 years [44]. Additionally, the C-index for the ASSURE trial group was found to be 0.812 (CI 0.775–0.848) [44]; however, there was no specific C-index reported for the development cohort, but authors mentioned superior discrimination compared to other models [44]. Although, a significant limitation of the ASSURE trial is the lack of external validation, making the appropriateness of extrapolating this model to independent patient populations unclear.

### 5.2. Leibovich Prognostic Model

For ccRCC, pRCC, and chrRCC, an important tool used to predict PFS and CSS is the 2018 Leibovich prognostic model. To create this model, 3633 patients with RCC from a single institution were retrospectively reviewed. A total of 2726 (75%) patients were diagnosed with ccRCC, 607 (17%) were diagnosed with pRCC and 222 (2%) were diagnosed with chrRCC [45]. Risk groups are divided up for PFS and CSS and by histology group. For PFS for ccRCC, patients can be scored from 0 to >15 [45]. Factors included are constitutional symptoms (no = 0 pts (points), yes = 1 pt, grade (1 = 0 pts, 2 = 1 pts, 3 = 2 pts, 4 = 3 pts), coagulative necrosis (no = 0 pts, yes = 2 pts), sarcomatoid differentiation (no = 0 pts, yes = 2 pts), tumor size (cm) (≤4 = 0 pts, >4 to ≥7 = 3 pts, >7 = 4 pts), perinephric or renal sinus fat invasion (no = 0 pts, yes = 1 pts), tumor thrombus (no = 0 pts, level 0 = 1 pts, level 1–4 = 2 pts), extension beyond kidney (no = 0 pts, yes = 2 pts), and nodal involvement (no nodal dissection or no = 0 pts, yes = 2 pt) [45]. The 5-year PFS ranges from 98% (95% CI = 97–98%) in patients with a ccRCC score of 0, 75% (95% CI = 72–77%) in a patient with a ccRCC score of 7 and 1% (95% CI = 0–1%) in patients with a ccRCC score of >15 [45].

To evaluate CSS, four additional variables are added so the score ranges from 0 to >18. The additional variables are age at surgery (year) (<60 = 0 pts, >60 pts = 1 pts), ECOG status (0 = 0 pts, >1 = 2 pts), adrenalectomy done (no = 0 pts, yes = 1 pts), and surgical margins (negative = 0 pts, positive = 1 pt) [45]. The range for CSS spans from 99% (95% CI = 99–99%) in patients with 0 points, 93% (95% CI = 92–94%) in patients with 7 points and 10% (95% CI = 7–13%) in patients with >18 points [45].

In pRCC, the variables that were independently associated with risk of progression or death from disease were grade (1–2, 3, or 4), perinephric or renal sinus fat invasion (no or yes), and tumor thrombus (no, level 0 or level 1–4) [45]. Risk group 1 included tumors that were grade 1–2, no fat invasion and no thrombus. Risk group 2 included grade 3, no fat invasion and no thrombus. Risk group 3 included grade 4 OR fat invasion OR any level thrombus. The 5-year PFS for pRCC risk group 1 is 97% (95% CI = 96–98%), risk group 2 is 89% (95% CI = 85–92%) and risk group 3 is 60% (95% CI = 49–69%) [45]. The 5-year CSS for pRCC risk group 1 is 99% (95% CI = 98–100%), risk group 2 is 95% (95% CI = 91–97%) and risk group 3 is 74% (95% CI = 59–84%) [45].

In chrRCC, the variables that were independently associated with risk of progression or death from disease were sarcomatoid differentiation (no or yes), perinephric or renal sinus fat invasion (no or yes), and nodal involvement (no/no nodal dissection or yes) [45]. Risk group 1 included carcinoma with no fat invasion, no sarcomatoid differentiation and no nodal involvement. Risk group 2 included fat invasion AND no sarcomatoid differentiation and no nodal involvement. Risk group 3 included sarcomatoid differentiation OR nodal involvement with or without fat invasion. The 5-year PFS for chrRCC risk group 1 is 94% (95% CI = 88–97%), risk group 2 is 71% (95% CI = 58–80%) and risk group 3 is 13% (95% CI = 0–28%) [45]. The 5-year CSS for chrRCC risk group 1 is 96% (95% CI = 95–98%), risk group 2 is 85% (95% CI = 79–89%) and risk group 3 is 49% (95% CI = 32–62%) [45].

### 5.3. GRade, Age, Nodes, and Tumors (GRANT) Risk Stratification Tool

The GRade, Age, Nodes, and Tumors (GRANT) risk stratification tool is another tool that aimed at stratifying risk of recurrence to aid in developing a prognosis in patients who had surgically treated pRCC [46]. There are four parameters for risk stratification with each of these variables equaling one point: Fuhrman grade 3–4, age >60, pathological nodal status 1–2 and pathological tumor size pT3b/c-4 [46]. Score of 0–1 places patients in the favorable group and a score of ≥2 places patients in the unfavorable group [46]. A study performed by Maffezzoli et al. in March 2025 analyzed GRANT risk stratification tool [47]. This study included 1942 patients treated with partial or radical nephrectomy for papillary RCC (pRCC) between 1984 and 2015. The study included only patients 18 or older with unilateral pRCC. The study looked at data including follow-up time, death from disease, center, age at surgery, sex, pRCC subtype, T classification, and lymph node involvement. Using these data points, they stratified patients using a three-group system (0–1 vs. two vs. 3–4 risk factors) in order to predict CSSl [47]. The study concluded that CSS correlated with GRANT group classification with group 1 (0–1 risk factors) having CSS of 93.2% (95% CI of 91.7–94.6%), group 2 (two risk factors) with CSS 60.8% (95% CI of 54.0–78.6%) and group 3 (3–4 risk factors) with CSS of 26.0% (95% CI of 15.7–42.9%) [47]. The GRANT risk stratification model demonstrated a concordance index of 0.73 in the original validation study [47]; however, several methodological considerations warrant acknowledgment. The reliance on historical registry data from the SEER database may limit generalizability to contemporary patient populations, given evolving treatment paradigms and patient demographics. Additionally, the retrospective study design introduces inherent susceptibility to selection bias, information bias, and unmeasured confounding, which may affect the model’s external validity and calibration in prospective clinical applications.

## 6. Adjuvant Therapy Patient Selection

The role of adjuvant immunotherapy in renal cell carcinoma has expanded significantly following the results of the pivotal KEYNOTE-564 trial [48], which demonstrated improved disease-free survival with adjuvant pembrolizumab in selected high-risk ccRCC patients after nephrectomy [48,49,50]. As a result, appropriate risk stratification has become essential to identify patients most likely to benefit while avoiding unnecessary toxicity in those with a low likelihood of recurrence. Current guideline-defined high-risk categories incorporate established anatomic and pathologic criteria, primarily rooted in the AJCC TNM staging system and tumor grade. Patients eligible for adjuvant therapy in KEYNOTE-564 include those with pT2 grade 4 or sarcomatoid differentiation, pT3 or pT4 disease of any grade, node-positive disease, or M1 with no evidence of disease (NED) status after complete metastasectomy [48,49,50]. All patients had clear cell histology and were ECOG 0-1. Within the trial, these were further organized into three prespecified risk cohorts: intermediate–high risk (pT2 grade 4/sarcomatoid or pT3 N0M0), high risk (pT4 or pTany with N1 disease), and M1 NED. These cohorts reflect distinct patterns of recurrence risk and allowed the trial to demonstrate consistent benefit across all three high-risk strata. These groups have been historically associated with substantial recurrence risk and were the populations from which KEYNOTE-564 derived its clinical benefit [48,49,50].

In addition, the ongoing LITESPARK-022 trial, which was presented at GUASCO 2026, evaluates pembrolizumab in combination with the HIF-2α inhibitor belzutifan using nearly identical risk classifications to KEYNOTE-564, including intermediate-high risk, high-risk, and M1-NED cohorts restricted to clear cell RCC with ECOG 0-1 performance status [51]. Early results suggest that intensified adjuvant therapy may further improve disease-free survival in this precisely risk-enriched postoperative population [51]. Final publication of LITESPARK-022 is eagerly awaited and the ALLIANCE Cooperative Group’s STRIKE trial (adjuvant pembro +/− tivo) is ongoing to test the efficacy of IO+TKI in the adjuvant setting.

Collectively, these trials demonstrate that evidence-based patient selection through rigorous risk stratification is fundamental to optimizing adjuvant therapy outcomes and identifying populations most likely to benefit from intervention. The evolution of prognostic models that integrate standardized, biologically relevant risk parameters will advance precision oncology in the adjuvant setting and refine both patient selection criteria and endpoint interpretation in subsequent immunotherapy trials.

## 7. Metastatic RCC Risk Models (Table A3)

### 7.1. Memorial Sloan Kettering Cancer Center Risk Model

The Memorial Sloan Kettering Cancer Center (MSKCC) risk model (“Motzer criteria”) aimed to predict outcomes in treatment naive patients with metastatic RCC. The risk model stratifies patients into three risk categories: favorable (0 points), intermediate (1–2 points), and poor risk (3–5 points) [52]. Patients receive one point for each of the following risk factors: Karnofsky performance status <80, elevated LDH greater than 1.5 times the upper limit of normal, anemia, corrected serum calcium >10, and time from initial diagnosis to start of systemic therapy <1 year [52]. Motzer et al. were foundational in establishing the MSKCC risk model where their study identified the five vital prognostic factors previously stated and their ability to stratify patients into risk categories [52]. Their study showed that median time to death was 20 months, 10 months and 4 months, respectively, for favorable, intermediate, and poor-risk patients [52]. In 2005, Mekhail et al. further validated and also attempted to expand the previous “Motzer criteria” to include prior radiotherapy and presence of hepatic, lung or nodal metastasis [53]. However, the addition of these two prognostic factors into the official MSKCC calculator was not widely adopted, and to this day, is not included in the risk MSKCC/Motzer score for metastatic RCC.

### 7.2. International Metastatic Renal Cell Carcinoma Database Consortium

The International Metastatic Renal Cell Carcinoma Database Consortium (IMDC) risk stratification tool uses six clinical and laboratory parameters to classify patients with metastatic RCC (mRCC) into favorable, intermediate, and poor-risk categories. This risk stratification tool was developed by Heng et al. in 2013 using the previous MSKCC risk stratification tool with the addition of neutrophil and platelet count [38]. Heng et al. specifically looked at patients with mRCC treated with vascular endothelial growth factor (VEGF) inhibitors [38]. The six risk parameters include: time from diagnosis to therapy <1 year, Karnofsky performance status <80%, hemoglobin below lower limit of normal, corrected calcium above upper limit of normal, absolute neutrophil count above upper limit of normal, platelet count above upper limit of normal. Patients are assigned to risk groups based on the number of risk factors present. Patients are considered favorable risk if no risk factors are present, intermediate risk if 1–2 risk factors are present, and poor-risk if 3–6 risk factors are present. The study found that favorable, intermediate and poor-risk groups had a 2-year overall survival of 75%, 53%, and 7%, respectively (log-rank *p* < 0.0001, C-index was 0.73) [38]. They also concluded that both neutrophils and platelets are above the upper limit of normal with independent adverse prognostic factors. Inclusion of these two additional risk factors helped to further stratify patients and aids in clinical decision making regarding care and treatment of patients with mRCC [38].

The IMDC has also since been validated in the immuno-oncology (IO) era. Yip et al. evaluated 687 patients with mRCC who were treated with one or more lines of immunotherapy from the IMDC database [54]. Patients were categorized into favorable, intermediate and poor-risk groups using the IMDC data. For patients who were treated with first-line immunotherapy, the median OS was not reached (NR) for any of the risk groups, but for patients treated with second-line immunotherapy, although, median OS was not reached for the favorable group, the median OS was 26.7 months (95% CI 20.6–48.5 months) in the intermediate-risk group and 7.4 months (95% CI 4.8–16.7) in the poor-risk group (*p* < 0.0001) [54]. For patients treated with third-line immunotherapy, median OS was reached in the favorable-risk (36.1 months, 95% CI 23.6-NR), intermediate-risk (28.2 months, 95% CI 12.0 months-NR), and poor-risk (11.1 months, 95% CI 4.5–39.1 months) groups (*p* = 0.016) [54].

More recently, in a retrospective cohort study published in the JAMA Network Open, Zarba et al. provided the first combined clinical and molecular validation of a “very favorable” subgroup within the IMDC favorable-risk category in metastatic renal cell carcinoma (mRCC) [55]. Using the IMDC database (January 2015–September 2024), 641 patients with IMDC favorable risk mRCC were stratified into tier 1 (very favorable), defined by Karnofsky performance status ≥ 90%, time from diagnosis to systemic therapy ≥ 3 years, and absence of brain, liver, or bone metastases (*n* = 176; 27.5%), and tier 2 (standard favorable) (*n* = 465; 72.5%) [55]. Tier 1 patients demonstrated a significantly longer median overall survival (OS) of 79.1 months (95% CI, 73.7 to not reached) compared with 54.5 months (95% CI, 45.5–67.7) in tier 2 (*p* < 0.001), with this survival advantage persisting across the entire four-tier IMDC risk spectrum (log-rank *p* < 0.001) [55]. Molecular profiling leveraging whole-exome sequencing, RNA sequencing, and PD-L1 immunohistochemistry from the IMmotion151 trial revealed that tier 1 tumors harbored a distinct biological signature: high PBRM1 alteration frequency (64.7%), low BAP1 alterations (8.8%), low PD-L1 positivity (21.9%), and enrichment for angiogenic transcriptomic clusters (59.6%) with markedly fewer immunogenic clusters (8.5%) [55].

This molecular profile translated into differential treatment responses: within the very favorable subgroup, dual immune checkpoint inhibitor therapy (IO-IO; ipilimumab plus nivolumab) was associated with significantly inferior outcomes, with a 2-year OS of only 73.1% and an ORR of 26.3%, compared with 89.1% and 63.3% for IO-VEGF combinations and 92.4% and 57.0% for VEGF-targeted therapy alone (IO-IO HR for death, 3.64; 95% CI, 1.49–9.06; *p* = 0.005) [55]. The authors conclude that this refined stratification has important implications for treatment selection and future clinical trial design in favorable-risk mRCC [55].

### 7.3. Selection Criteria for Cytoreductive Nephrectomy Score

Likewise, the selection criteria for cytoreductive nephrectomy (SCREEN) score was developed by Abel et al. in 2024 as a prognostic model to help improve patient selection for cytoreductive nephrectomy (CN) [56]. The study evaluated 914 patients with mRCC treated with upfront CN at five institutions between 2006 and 2017. Seven variables helped determine prognostic score: three or more metastatic sites, total metastatic tumor burden ≥5 cm, bone metastasis, systemic symptoms, low serum hemoglobin, low serum albumin, and neutrophil/lymphocyte ratio ≥4 [56]. When compared with the IMDC risk stratification of favorable, intermediate and poor-risk categories, the SCREEN score was shown to have higher predictive accuracy for first-year mortality and may be more useful in careful patient selection for CN [56].

Collectively, these risk models underscore the importance of integrating performance status, disease burden, and systemic inflammatory markers when stratifying patients with metastatic RCC. While MSKCC and IMDC remain widely adopted in clinical trials and real-world practice, emerging models such as SCREEN demonstrate the field’s ongoing effort to improve prognostic accuracy and treatment selection. Continued refinement of these tools will be essential as novel, targeted and immunotherapeutic approaches reshape the metastatic RCC landscape.

## 8. Molecular and Genomic Biomarkers as Prognostic Tools

### 8.1. SETD2 and Tumor-Infiltrating B Lymphocyte Biomarkers

Given the complexity of prior prognostic models, as well as lack of obvious consensus on which is most beneficial, the field is now shifting toward identifying possible biomarkers to aid in risk stratification, prognostication and guidance for targeted therapies. In May of 2025, Wong et al. provided a comprehensive review of genomic, protein, immunologic, and radiologic biomarkers currently being investigated as potential prognostic and therapeutic indicators [57]. Specifically, there has been a great interest in chromosome 3p genes given its link to Von Hippel–Lindau most commonly linked with ccRCC. Additionally, the authors highlighted specific interest in SET domain containing 2 (SETD2), given its critical role in maintaining genomic stability via homologous recombination repair, and its high mutational frequency in RCC [57,58]. Other genes have been implicated in the tumorigenesis of RCC and been studied as prognostic indicators, although few have been validated and widely used (Table 4) [57,59]. Furthermore, a number of proteins, such as CRP [60] and secretagogin (SCGN) [61], as well as immune biomarkers, such as tumor-infiltrating B lymphocytes (TIBs) [62] and neutrophil-to-lymphocyte ratio (NLR) [63], have been shown to have prognostic associations with cancer outcomes in RCC (Table 5 and Table A1) [57].

### 8.2. ARL4C, ECT2, SOD2 and STEAP3 Biomarkers

Likewise, Kubota et al. conducted an immunohistochemical analysis of four specific biomarkers—ARL4C, ECT2, SOD2, and STEAP3—in primary RCC tissue obtained post-nephrectomy [64]. They further investigated the expression of these individual biomarkers in relation to cancer-specific survival [64]. The authors grouped the samples into high expression if tumor staining intensity was greater than or equal to that of normal proximal tubules, and lower if staining intensity was less than that of normal proximal tubules [64]. They found that the 5 year CSS for high- and low-ARL4C groups were 51.2% and 95.1%, respectively (*p* < 0.001, Chi-square value = 28.11) [64]. The 5-year CSS was 67.7% and 84.4% for the high- and low-ECT2 groups, respectively (*p* = 0.023, chi-square value = 5.18), 55.8% and 85.0% for the SOD2 groups (*p* = 0.005, chi-square value = 8.02) and 46.9% and 82.6% for STEAP3 (*p* < 0.001, chi-square value = 24.35) [64]. Additionally, they found that “metastasis at diagnosis (HR = 9.17, 95% CI = 1.45–57.83, *p* = 0.018), T3/4 (HR = 12.00, 95% CI = 1.50–96.30, *p* = 0.019) and high ARL4C expression (HR = 10.98, 95% CI = 1.77–68.02, *p* = 0.010) were significant risk factors for worse CSS” [64]. This study ultimately emphasized the need to further study tumor biomarkers as identification of these biomarkers are effective in predicting CSS and will aid in patient counseling, prognostication, and potential gene-targeted therapies.

### 8.3. Bio-miR Biomarkers

Bio-miR, is the combined expression of nine microRNA used as a prognostic biomarker that Pinto-Marin et al. studied to stratify localized clear cell RCC into low- and high-risk groups [65]. This study characterized high- and low-risk groups in patients with localized clear cell renal cell carcinoma (ccRCC) using a nine-microRNA expression signature derived from tumor tissue. Patients were classified as high or low risk for relapse based on the combined expression profile of these nine microRNAs, which was validated across multiple independent cohorts [65]. The 5-year disease-free survival (DFS) was 87.1% in the low-risk group versus 54.2% in the high-risk group (*p* = 0.0086; HR = 3.58; 95% CI: 1.37–8.3) [65]. A few various groups were used to validate this prognostic model which confirmed similar results. A UK cohort of 75 patients had a 5-year DFS of 94% vs. 62% in low-risk vs. high-risk patients (HR = 7.14, *p* = 0.001) [65]. A total of 161 patients were analyzed in a Spanish cohort and it was found that 5-year DFS was 82.9% vs. 58.7% for low vs. high risk (HR = 2.46, *p* = 0.0013) [65]. The 5-year OS rate is 72.7% vs. 44.5% for low vs. high risk in a The Cancer Genome Atlas cohort of 147 patients (HR = 2.43, *p* = 0.0012) [65].

### 8.4. KIM-1 Biomarker

A newly studied glycoprotein biomarker, kidney injury molecule-1 (KIM-1) shows great promise as a prognostic guide and possible indicator for microscopic residual disease. One study of the ASSURE trial evaluated KIM-1 levels after nephrectomy and was associated with decreased disease-free survival with a survival time ratio of 0.56 for 75th percentile of KIM-1/25th percentile of KIM-1 (95% CI 0.42–0.73, *p* < 0.001) [66]. When using the ASSURE patients, when KIM-1 was included in models such as SSIGN and UISS models, the concordance was higher, with 0.57 vs. 0.43 for SSIGN (*p* = 0.052) and 0.60 vs. 0.40 for UISS (*p* = 0.0005) [66]. These studies continue to arise and highlight the need for more discovery into tumor biomarkers, as these, in combination with traditional risk stratification, may be the key to unlocking better patient-specific counseling and treatments.

### 8.5. Soluble MAdCAM-1 as a Biomarker

A recent study by Alves Costa Silva et al. published in January 2026 evaluated 1051 patients across three independent cohorts: JAVELIN Renal 101 (phase III trial comparing avelumab plus axitinib versus sunitinib in treatment-naïve mRCC), SURF (sunitinib cohort), and NIVOREN (nivolumab after TKI failure) [67]. In the JAVELIN Renal 101 cohort (612 patients with available samples), patients with sMAdCAM-1 > 180 ng/mL had significantly superior outcomes: median PFS of 13.9 months versus 8.4 months (*p* < 0.01) and 18-month OS rate of 84.2% versus 68.1% (*p* < 0.01) [67]. These associations remained significant after adjusting for IMDC risk groups, and the prognostic model incorporating IMDC plus sMAdCAM-1 improved the AUC at 18 months from 0.68 to 0.72 (*p* = 0.01) [67]. Importantly, the prognostic value was independent of treatment arm, suggesting sMAdCAM-1 predicts outcomes to both ICI-based combinations and TKI monotherapy [67]. The prognostic value was validated in the SURF and NIVOREN cohorts for overall survival. In the NIVOREN cohort (212 patients), low sMAdCAM-1 was associated with dramatically worse outcomes: median OS of 13.3 months versus 25.3 months (HR 2.97, *p* < 0.0001) and median PFS of 2.6 months versus 5.2 months (HR 1.92, *p* < 0.0001) [67]. The authors propose that sMAdCAM-1 could serve as a biomarker-guided tool to identify patients who might benefit from microbiota-targeted interventions, such as fecal microbiota transplantation, Akkermansia muciniphila supplementation, or IL-17A neutralization. The prognostic value extends beyond RCC, as sMAdCAM-1 also predicted OS in 381 patients with metastatic lung and bladder cancer treated with ICIs [67].

## 9. Barriers to Biomarker Implementation

The translation of these and other emerging biomarkers from bench to bedside faces substantial economic and logistical barriers that have impeded their uniform adoption across clinical practice. Next-generation sequencing (NGS), the platform underlying most genomic biomarker assays, carries average costs ranging from approximately $1269 to $2058 per test in the United States, with considerable variability by region, payer, and panel size [68]. Coverage and reimbursement remain inconsistent; Medicare claim denials for cancer-related NGS testing have been documented, and reimbursement policies vary substantially across payers and geographies [69]. In Europe, access to advanced biomolecular technologies is highly heterogeneous, with the most important barriers identified as reimbursement of the test itself (59% of respondents) and availability of a suitable matched therapy (48%) [70].

Beyond cost, additional barriers include: the lack of prospective, multi-institutional validation for most candidate biomarkers; the absence of standardized assay protocols and reproducibility data; variable genomic literacy among treating physicians; limited access to NGS infrastructure outside academic centers; and the challenge of tissue availability, particularly for liquid biopsy approaches that remain investigational [71]. A recent consensus initiative from the International Kidney Cancer Symposium emphasized the need for rigorous methodology, collaborative infrastructure, prospective data collection, and a focus on clinically translatable biomarkers to advance personalized care [72]. Until these barriers are systematically addressed, the integration of molecular biomarkers into routine RCC risk stratification will remain aspirational rather than standard practice, and clinicians must continue to rely on validated clinical-laboratory models while remaining attuned to the rapidly evolving molecular landscape.

## 10. Advances in Variant Histology

The classification of variant histology renal cell carcinoma (VH-RCC) has undergone a paradigm shift with the publication of the 2022 World Health Organization (WHO) 5th Edition Classification of Tumors of the Urinary System and Male Genital Organs [73,74]. Most notably, the WHO 2022 classification introduced a new category of molecularly defined renal carcinomas, encompassing seven entities—TFE3rearranged, TFEB-altered, ELOC-mutated, fumarate hydratase (FH)-deficient, succinate dehydrogenase (SDH)-deficient, ALK-rearranged, and SMARCB1-deficient renal medullary carcinoma—that are now classified primarily by their underlying molecular alterations rather than morphologic appearance alone [74,75]. This molecular-integrated approach has had immediate implications for the reclassification of papillary RCC, with the elimination of the traditional type 1/type 2 subcategorization owing to considerable overlap and limited clinical utility, and the recognition that many tumors previously designated as type 2 papillary RCC represent distinct molecular entities such as FH-deficient RCC and MiTF family translocation carcinomas [75,76]. Additional morphologically defined entities, including eosinophilic solid and cystic RCC and a new category of “other oncocytic tumors” harboring TSC/mTOR pathway alterations, have further expanded the taxonomic landscape [74,77]. As the integration of genomic profiling with histopathologic assessment continues to evolve, with emerging evidence identifying subtype-specific therapeutic vulnerabilities in rare molecularly characterized tumors, the accurate classification of VH-RCC will be increasingly essential for guiding precision medicine approaches and designing appropriately stratified clinical trials.

## 11. Salvage Therapy

Risk stratification in the salvage setting for renal cell carcinoma (RCC) remains an evolving and incompletely standardized domain. The International Metastatic Renal Cell Carcinoma Database Consortium (IMDC) model, originally developed and validated for first-line targeted therapy, has been subsequently validated in patients receiving second-line targeted therapy following progression on first-line VEGFR-directed agents [78]. This demonstrated superior prognostic accuracy compared with the Memorial Sloan Kettering Cancer Center (MSKCC) model in this context [78]. Notably, approximately 40% of patients experience a shift in IMDC prognostic category between first- and second-line therapy, and these transitions have been shown to independently predict clinical outcomes and may inform the selection of subsequent therapeutic class [79].

Additional prognostic factors identified in the salvage setting include the neutrophil-to-lymphocyte ratio, tumor burden at the time of progression, duration of first-line progression-free survival, and Karnofsky performance status [78]. More recently, emerging models such as the ACL (albumin, C-reactive protein, and lactate dehydrogenase) score have demonstrated comparable or superior discriminatory performance relative to the IMDC and MSKCC models in patients receiving second-line therapy after first-line immune checkpoint inhibitor combinations [80]. Furthermore, early tumor shrinkage on first-line therapy and patterns of progressive disease—such as the development of new lesions versus growth of existing lesions—have been proposed as dynamic prognostic indicators that may refine risk assessment at the time of salvage therapy initiation [79,81]. Despite these advances, the absence of prospectively validated, molecularly integrated prognostic tools specifically designed for the post-immunotherapy salvage setting represents a critical unmet need, particularly as the treatment landscape continues to shift toward immune checkpoint inhibitor-based first-line regimens.

## 12. Conclusions

While numerous prognostic and risk stratification models exist, these standard models do not incorporate tumor biology. Incorporation of tumor biology is a promising area for improving model performance in the future. The urgent need for a unified, evidence-based prognostic tool tailored to specific pathologic and histologic subtypes is clear. The heterogeneity of RCC at the molecular level fundamentally limits the utility of clinical–pathologic models alone. Current risk models, which rely primarily on TNM stage, grade, performance status, and laboratory parameters, do not directly reflect tumor biology and therefore cannot capture this molecular heterogeneity. As a result, patients within the same clinical risk category may experience vastly different outcomes, undermining the precision needed for individualized treatment decisions. Biomarkers—including circulating tumor DNA (ctDNA), methylated DNA, AI-based radiomics, and tissue-based molecular signatures such as the Bio-miR score and SSPN score—represent critical opportunities to identify high-risk patients with greater precision. As biomarkers continue to show promise, they represent a critical opportunity to better identify high-risk patients and enable more precise, individualized care. Some prognostic tools like IMDC and SSIGN rely on clinical and pathologic variables that act as indirect surrogates of tumor biology and do not account for molecular heterogeneity across RCC subtypes. As a result, patients within the same risk category may have substantially different outcomes, underscoring the need for integrated molecular–clinical–pathologic prognostic models.

## 13. Future Directions

The current landscape of risk stratification in RCC is complex, with numerous prognostic tools spanning clinical-laboratory models, pathological scoring systems, and emerging genomic and molecular biomarkers—none of which has achieved universal dominance. In the metastatic setting, the IMDC and MSKCC risk models remain the most widely utilized, while localized disease relies on integrated systems such as UISS and SSIGN, supplemented by evolving molecular signatures including somatic mutations (BAP1, PBRM1, TP53), transcriptomic panels, and circulating tumor DNA [82,83,84,85], (Table A2). Notably, the role of risk stratification in the neoadjuvant setting remains largely unexplored, representing a significant gap in the literature and a promising avenue for future investigation. As neoadjuvant immunotherapy and combination regimens gain traction in clinical trials, the development and validation of predictive biomarkers tailored to this treatment context will be essential for optimizing patient selection and therapeutic outcomes. Similarly, there remains a lack of well-validated scoring systems for use in the salvage setting. Given this heterogeneous landscape, it is important that oncologists and urologists maintain familiarity with the array of available risk stratification systems and select a few that they use routinely. When selecting a risk schema, it is imperative to apply the appropriate tool to the appropriate patient population—organ confined vs. metastatic, clear cell vs. non-clear cell, etc.—while being mindful of each system’s limitations. We eagerly await the ASCO Clinical Practice Guideline on perioperative systemic therapy for RCC, which is expected to be published later this year.

## Figures and Tables

**Table 1:** Differences between renal cell carcinoma AJCC primary tumor staging editions [6,7].

**Category**	**6th Edition (2002)**	**7th Edition (2010)**	**8th Edition (2017)**
Tx	Primary tumor cannot be assessed		
T0	No evidence of primary tumor		
T1	Tumor ≤ 7 cm, limited to kidney		
T1a	Tumor ≤ 4 cm, limited to kidney		
T1b	Tumor > 4 cm but ≤7 cm, limited to kidney		
T2	Tumor > 7 cm, limited to kidney		
T2a	—	Tumor > 7 cm but ≤10 cm, limited to kidney	
T2b	—	Tumor > 10 cm, limited to kidney	
T3	Tumor extends into major veins or invades adrenal gland or perinephric tissues, but not beyond Gerota’s fascia	Tumor extends into major veins or perinephric tissues, but not into ipsilateral adrenal gland and not beyond Gerota’s fascia	
T3a	Tumor directly invades adrenal gland or perirenal/renal sinus fat, not beyond Gerota’s fascia	Tumor invades renal vein or segmental branches, or perirenal/renal sinus fat, not beyond Gerota’s fascia	
T3b	Tumor extends into renal vein or vena cava below diaphragm	Tumor extends into vena cava below diaphragm	
T3c	Tumor extends into vena cava above diaphragm or invades vena cava wall	Tumor extends into vena cava above diaphragm or invades wall	
T4	Tumor invades beyond Gerota’s fascia	Tumor invades beyond Gerota’s fascia (including ipsilateral adrenal gland)	
Nx	Regional lymph nodes cannot be assessed		
N0	No regional lymph node metastasis		
N1	Metastasis in a single regional lymph node	Metastasis in regional lymph node(s)	
N2	Metastasis in >1 regional lymph node	—	—
M0	No distant metastasis		
M1	Distant metastasis		
**Stage**	**6th Edition (2002)**	**7th Edition (2010)**	**8th Edition (2017)**
Stage I	T1 N0 M0	Same	Same
Stage II	T2 N0 M0	Same	Same
Stage III	T1–2 N1 M0; T3 N0–1 M0	T1–2 N1 M0; T3 N0–1 M0	Same
Stage IV	T4 any N M0; any T N2 M0; any T any N M1	T4 any N M0; any T any N M1	Same

**Table 2:** AUA RCC risk stratification and recurrence rates [11,12,14].

Risk Category	Clinicopathologic Variables	Estimated Recurrence Rate
Low risk	pT1; Grade 1–2	6.4–15.4%
Intermediate risk	pT2 (any grade)	20–32%
High risk	pT3 (any grade)	~50%
Very high risk	pT4; pN1; sarcomatoid differentiation; rhabdoid differentiation; macroscopic positive margin	>65%

**Table 3:** Comparison of 8E AJCC prognostic groups to other proposed classification schemes [19].

Stage	AJCC 8th Edition	Shao et al. [21] Proposed Staging	Yu et al. [22] Proposed Staging	Integrated Proposed Staging
I	T1 N0 M0	Ia: T1 N0 M0; Ib: T2 N0 M0	T1 N0 M0	T1 N0 M0
II	T2 N0 M0	T3 N0 M0	T2 N0 M0	T2 N0 M0
III	T1–2 N1 M0; T3 N0 any M0	T1–3 N1 M0; T4 N0 M0	T3 N0 M0	T3 N0 M0
IV	T4 any N M0; any T any N M1	T4 N1 M0; any T any N M1	T1–3 N1 M0; T4 any N M0; any T any N M1	IVa: T3 N1 M0; T3 N0 M1; T4 N0 M0; IVb: T4 N1 M0; T4 N0 M1; T4 N1 M1

**Table 4:** Genes involved in RCC tumorigenesis [59].

Gene	Chromosomal Location	Primary Function/Pathway	Associated Clinical Features/Notes
*SDHB*	1p36.13	Mitochondrial enzyme; cellular metabolism	Associated with aggressive tumors
*FH*	1q43	Tricarboxylic acid (TCA) cycle enzyme; metabolism	Mutation associated with hereditary leiomyomatosis and renal cell carcinoma (HLRCC)
*MITF*	3p13	Transcription factor	Associated with translocation-related tumors
*BAP1*	3p21.1	SWI/SNF chromatin remodeling complex	Associated with rapid tumor growth
*VHL*	3p25.3	VHL–HIF–VEGF pathway; angiogenesis and cellular growth	Altered in ~90% of renal cell carcinoma cases
*TFEB*	6p21.1	Transcription factor	Associated with translocation-related tumors
*MET*	7q31.2	Tyrosine kinase; angiogenesis and tumor growth	Associated with papillary renal cell carcinoma
*TSC1*	9q34.13	Hamartin protein; regulation of cell growth	Mutation associated with tuberous sclerosis complex (TSC)
*PTEN*	10q23.31	Phosphatase; PIP3 regulation	Mutation associated with Cowden syndrome
*SDHD*	11q23.1	Mitochondrial enzyme; cellular metabolism	Associated with aggressive tumors
*TSC2*	16p13.3	Tuberin protein; regulation of cell growth	Mutation associated with tuberous sclerosis complex (TSC)
*FLCN*	17p11.2	Cellular metabolism	Mutation associated with Birt–Hogg–Dubé syndrome
*TFE3*	Xp11.23	Transcription factor	Associated with translocation-related tumors

**Table 5:** Summary of proteins discussed for use as biomarkers in RCC.

Biomarker	Molecular Pathway/Function	Association in RCC
CRP	Acute-phase inflammatory protein involved in modulation of the immune response	Elevated levels are associated with worse prognosis
PDZK1	Scaffolding protein; inhibits platelet-derived growth factor (PDGF) signaling and cell proliferation	Increased expression is associated with enhanced sensitivity to sunitinib
ZNF582	Transcription factor involved in regulation of cell proliferation, apoptosis, and metabolism	Decreased expression is associated with worse prognosis
SCGN	Calcium-binding protein involved in gene expression, cell growth, and protein synthesis	Upregulation is associated with improved prognosis
STAT3	Signaling protein involved in signal transduction and gene expression	Downregulation is associated with improved prognosis

Abbreviations: PDGF, platelet-derived growth factor; ZNF582, Zinc finger protein 582; SCGN, secretagogin.

## Data Availability

No original data were generated in the conduct of this study. All tables represent a synthesis of data extracted from the previously published literature. Readers are directed to the cited references for primary source material.

## Appendix A

**Table A1:** Summary of the immunologic profiles discussed for use as biomarkers in RCC.

Biomarker	Study Population	Association with RCC
TIBs	Metastatic clear cell RCC patients treated with anti-PD-1 immune checkpoint inhibitors (e.g., pembrolizumab or tislelizumab)	High TIB infiltration is associated with lower objective response rate (ORR) and poorer overall survival (OS) and progression-free survival (PFS)
NLR	Metastatic RCC patients receiving nivolumab plus ipilimumab	Higher neutrophil-to-lymphocyte ratio is associated with worse survival outcomes
PSMD2	Advanced RCC patients treated with immune checkpoint inhibitor (ICI) and tyrosine kinase inhibitor (TKI) combination therapy	Increased expression is associated with reduced PFS and increased resistance to ICI/TKI combination therapy
PD-1 Treg/CD8^+^ ratio	Metastatic clear cell RCC patients treated with nivolumab	Increased ratio is associated with shorter PFS and OS

Abbreviations: TIBs, tumor-infiltrating B lymphocytes; ICI, immune checkpoint inhibitor; NLR, neutrophil-to-lymphocyte ratio; PSMD2, proteasome 26S subunit non-ATPase 2; TKI, tyrosine kinase inhibitor; Treg, regulatory T cells.

**Table A2:** Summary of risk stratification tools for locally advanced renal cell carcinoma.

Histology	Model	Variables	Risk Category	Definition/Score	Outcomes	C-Indexes (CSS/ DSS/OS)	Strengths	Weakness
Clear Cell RCC	5 microRNA signature	Combined expression of five microRNAs	Low risk	—	5-year DFS: 8li7.1–94%; 5-year OS: 72.7%	0.70–0.80	Potential for liquid biopsy application Therapeutic target identification Ability to substratify within existing risk groups	Not incorporated into any guidelines Extreme heterogeneity with no overlap Small cohorts No prospective trial validation Cost barriers
			High risk	—	5-year DFS: 54.2–58.7%; 5-year OS: 44.5%		Potential to refine adjuvant therapy selection	Binary risk stratification only
	SSIGN score	TNM stage; tumor size; nuclear grade; tumor necrosis; sarcomatoid features; cystic architecture; multifocality; surgical margin status	Group 1	Score 0–1	5-year CSS: 99.4%	0.82–0.9	Strong retrospective discriminatory performance Simplicity/accessibility Validated with routine pathology Durability overtime Granular risk stratification Recommended by major guidelines (EAU)	Restricted to clear cell RCC histologic type only Postoperative only tool No incorporation of clinical variables Significant decline in prospective validation Inability to further stratify intermediate risk patients
			Group 2	Score 2	5-year CSS: 94.8%			
			Group 3	Score 3	5-year CSS: 87.8%			
			Group 4	Score 4	5-year CSS: 79.1%			
			Group 5	Score 5	5-year CSS: 65.4%			
			Group 6	Score 6	5-year CSS: 54.0%			
			Group 7	Score 7	5-year CSS: 41.0%			
			Group 8	Score 8	5-year CSS: 23.6%			
			Group 9	Score 9	5-year CSS: 19.6%			
			Group 10	≥10	5-year CSS: 7.4%			
	UISS	TNM stage; Fuhrman grade; ECOG performance status	Low risk (non-metastatic)	—	5-year DFS: 91.1%	0.56–0.84	Histology-agnostic capability Incorporation of clinical performance status	Poor identification of high-risk patients Less accurate in metastatic disease Reliance on Fuhrman grading
			Intermediate risk	—	5-year DFS: 80.4%		Dual applicability to localized and metastatic disease	Does not incorporate pathological variables
			High risk	—	5-year DFS: 54.7%		Extensive multicenter validation Recommended by major guidelines (EAU)	
	Leibovich score	Constitutional symptoms; tumor grade; coagulative necrosis; sarcomatoid differentiation; tumor size; perinephric/sinus fat invasion; tumor thrombus; extra-renal extension; renal involvement	Group 1	0 points	5-year PFS: 98%; CSS: 99%	0.79–0.88	High discriminatory performance for ccRCC Histology-specific models Extensive interval validation	Single institution derivation Does not incorporate clinical variables Complexity compared to simpler models Lower performance in non-clear cell histologies
			Group 2	7 points	5-year PFS: 75%; CSS: 93%		Validated in diverse racial populations	Does not incorporate molecular or genomic data
			Group 3	≥15 (PFS) or >18 (CSS)	5-year PFS: 1%; CSS: 10%		Recommended by EAU guidelines	Postoperative only tool
Papillary RCC	GRANT score	Tumor grade; age > 60 years; nodal status; tumor size	Group 1	0–1 risk factors	5-year CSS: 93.2%	0.65–0.73	Simplicity Histology-agnostic capability	Reliance on Fuhrman grade Vulnerability to TNM staging changes
			Group 2	2 risk factors	5-year CSS: 60.8%		Validated in large population	Does not incorporate venous tumor thrombus/tumor necrosis
			Group 3	3–4 risk factors	5-year CSS: 26.0%		Incorporation of age Recommended in EAU guidelines	Does not incorporate molecular or genomic data
	Leibovich score	Same variables as above	Group 1	0 points	5-year PFS: 97%; CSS: 99%	0.70–0.92	Histology-specific design	Lowest c-indexes across pRCC risk tools
			Group 2	7 points	5-year PFS: 89%; CSS: 95%		Validated across diverse populations Simplicity	Markedly reduced performance in black patients
			Group 3	≥15 (PFS) or >18 (CSS)	5-year PFS: 60%; CSS: 74%		Recommended by EAU guidelines	Does not incorporate molecular or genomic data Does not incorporate venous tumor thrombus
Chromophobe RCC	Leibovich score	Same variables as above	Group 1	0 points	5-year PFS: 94%	0.55–0.78	Only histology-specific model for chRCC	Does not incorporate coagulative necrosis
			Group 2	7 points	5-year CSS: 96%; 5-year PFS: 71%		Validated across diverse populations Captures key prognostic values	Small cohort with single institution bias Does not incorporate sarcomatoid differentiation
			Group 3	≥15 (PFS) or >18 (CSS)	5-year PFS: 13%; 5-year CSS: 49%		Recommended in EAU guidelines	No CSS developed due to low event/death rates

Abbreviations: RCC, renal cell carcinoma; DFS, disease-free survival; PFS, progression-free survival; OS, overall survival; CSS, cancer-specific survival; UISS, University of California Los Angeles Integrated Staging System; ECOG, Eastern Cooperative Oncology Group.

**Table A3:** Summary of risk stratification tools for metastatic renal cell carcinoma.

Model	Variables	Risk Category	Definition	Outcomes
MSKCC Risk Model	Karnofsky performance status <80%; lactate dehydrogenase >1.5× upper limit of normal; anemia; corrected serum calcium >10 mg/dL; time from diagnosis to systemic therapy <1 year	Favorable	0 risk factors	Median OS: 20 months
		Intermediate	1–2 risk factors	Median OS: 10 months
		Poor	3–5 risk factors	Median OS: 4 months
IMDC Risk Model	Time from diagnosis to treatment <1 year; Karnofsky performance status <80%; hemoglobin below lower limit of normal; corrected calcium above upper limit of normal; absolute neutrophil count above upper limit of normal; platelet count above upper limit of normal	Favorable	0 risk factors	2-year OS: 75%
		Intermediate	1–2 risk factors	2-year OS: 53%
		Poor	3–6 risk factors	2-year OS: 7%
SCREEN Score	≥3 metastatic sites; total metastatic tumor burden ≥5 cm; bone metastases; systemic symptoms; low hemoglobin; low serum albumin; neutrophil-to-lymphocyte ratio ≥4	Favorable	0–1 risk factors	Median OS: 120 months
		Intermediate	2–3 risk factors	Median OS: 27 months
		Poor	≥4 risk factors	Median OS: 26 months

Abbreviations: MSKCC—Memorial Sloan Kettering Cancer Center, IMDC—International Metastatic Renal Cell Carcinoma Database Consortium, SCREEN—selection criteria for cytoreductive nephrectomy, OS—Overall Survival.
